# Synthesis and preliminary preclinical evaluation of fluorine-18 labelled isatin-4-(4-methoxyphenyl)-3-thiosemicarbazone ([^18^F]4FIMPTC) as a novel PET tracer of P-glycoprotein expression

**DOI:** 10.1186/s41181-018-0046-z

**Published:** 2018-09-21

**Authors:** Joost Verbeek, Jonas Eriksson, Stina Syvänen, Marc Huisman, Robert C. Schuit, Carla F. M. Molthoff, Rob A. Voskuyl, Elizabeth C. de Lange, Adriaan A. Lammertsma, Albert D. Windhorst

**Affiliations:** 1 Department of Radiology & Nuclear Medicine, VU University Medical Center, P.O. box 7057, 1007 MB Amsterdam, The Netherlands; 2 Division of Pharmacology, LACDR, Leiden University, Leiden, The Netherlands; 3 Present Address: Department of Public Health and Caring Sciences, Uppsala University, Uppsala, Sweden; 4 Stichting Epilepsie Instellingen Nederland, SEIN, Heemstede, The Netherlands

**Keywords:** P-glycoprotein, P-gp inhibitor, [^18^F]4FIMPTC, Radiofluorination, [^18^F]-fluorisatin

## Abstract

**Background:**

Several P-glycoprotein (P-gp) substrate tracers are available to assess P-gp function in vivo, but attempts to develop a tracer for measuring expression levels of P-gp have not been successful. Recently, (Z)-2-(5-fluoro-2-oxoindolin-3-ylidene)-*N*-(4-methoxyphenyl)hydrazine-carbothioamide was described as a potential selective P-gp inhibitor that is not transported by P-gp. Therefore, the purpose of this study was to radiolabel two of its analogues and to assess their potential for imaging P-gp expression using PET.

**Results:**

[^18^F]2-(4-fluoro-2-oxoindolin-3-ylidene)-*N*-(4-methoxyphenyl)hydrazine-carbothioamide ([^18^F]**5**) and [^18^F]2-(6-fluoro-2-oxoindolin-3-ylidene)-N-(4-methoxyphenyl)hydrazine-carbothioamide ([^18^F]**6**) were synthesized and both their biodistribution and metabolism were evaluated in rats. In addition, PET scans were acquired in rats before and after tariquidar (P-gp inhibitor) administration as well as in P-gp knockout (KO) mice.

Both [^18^F]**5** and [^18^F]**6** were synthesized in 2–3% overall yield, and showed high brain uptake in ex vivo biodistribution studies. [^18^F]**6** appeared to be metabolically unstable in vivo, while [^18^F]**5** showed moderate stability with limited uptake of radiolabelled metabolites in the brain. PET studies showed that transport of [^18^F]**5** across the blood-brain barrier was not altered by pre-treatment with the P-gp inhibitor tariquidar, and uptake was significantly lower in P-gp KO than in wild-type animals and indeed transported across the BBB or bound to P-gp in endothelial cells.

**Conclusion:**

In conclusion, [^18^F]**5** and [^18^F]**6** were successfully and reproducibly synthesized, albeit with low radiochemical yields. [^18^F]**5** appears to be a radiotracer that binds to P-gp, as showed in P-gp knock-out animals, but is not a substrate for P-gp.

## Background

The blood-brain barrier (BBB) is a tightly connected cell layer. Its main function is to protect the brain from toxic substances by regulating their transfer between brain and blood. Transfer across the BBB is possible either through passive diffusion or through active influx and efflux (de Vries et al., 2005; Pike, 2009). One of the main proteins involved in active efflux from the brain is P-glycoprotein (P-gp). P-gp is an ATP-dependent 170–180 kDa transmembrane glycoprotein and, being located at the luminal side of the BBB, it lowers brain concentrations of its substrates. P-gp is involved in the transport of many small molecules from the brain back into the circulation (de Vries et al., 2005; Pike, 2009) and, therefore, it plays a major role in the brain pharmacokinetics of many drugs. In addition, changes in P-gp function are associated with several diseases, such as HIV (Kim et al., 1998), depression (Szabo et al., 1999) and temporal lobe epilepsy (Löscher & Sills, 2007; Luna-Tortos et al., 2008; Löscher & Potschka, 2002).

The function of P-gp can be studied using positron emission tomography (PET) (Luurtsema et al., 2010; Syvänen & Eriksson, 2013). To date, several PET tracers have been developed based on known P-gp substrates to image cerebral P-gp function, such as *(R)*-[^11^C]verapamil (Elsinga et al., 1996; Luurtsema et al., 2005) and [^11^C]desmethyl loperamide (Zoghbi et al., 2008). Cerebral uptake of these substrate tracers is inversely related to P-gp function. Although, subjects with decreased P-gp function show a higher brain uptake of a substrate tracer, small but significant differences were observed in practice (van Assema et al., 2012; van Assema et al., 2010). This is probably due to the high capacity of P-gp and the relatively high noise levels associated with P-gp substrate tracers, however the clinical relevance is limited by the small effects. In addition, an increase in P-gp function, as proposed in pharmacoresistant epilepsy, is considerably more difficult to assess using P-gp substrate tracers, as it requires quantification of a decrease in tracer uptake from an already low level (Deo et al., 2014). In addition, Deo et al. (Deo et al., 2014) showed that in AD patients it was difficult to detect reduced P-gp function with *(R)*-[^11^C]verapamil, which was only successful when correcting K_1_ values for cerebral blood flow, illustrating the difficulties when using a substrate tracer for measuring expression of P-gp. In addition van Assema et al. found a small increase (*R*)-[^11^C]Verapamil BP_ND_ in the brain of patients with Alzheimer’s disease as compared with values in healthy age-matched controls, which was however difficult to analyse due to the low brain concentrations of (*R*)-[^11^C]Verapamil (van Assema et al., 2012).

An alternative approach to image P-gp upregulation with PET is the use of tracers, which are weak to moderate P-gp substrates such as [^11^C]phenytoin (Verbeek et al., 2012) and [^11^C]quinidine (Syvänen et al., 2013). These tracers show higher brain uptake than [^11^C]verapamil (Verbeek et al., 2012) and, therefore, it may be easier to measure reduced uptake as a result of P-gp upregulation. On the other hand, these tracers are weaker substrates for P-gp and show less pronounced blocking effects.

P-gp substrates are low affinity inhibitors, e.g. quinidine will inhibit P-gp function at sufficiently high doses. Under these conditions, most inhibitors would be expected to be substrates at low concentrations as indeed observed for known P-gp inhibitors such as [^11^C]laniquidar (Luurtsema et al., 2009), [^11^C]elacridar (Dorner et al., 2009) and [^11^C]tariquidar (Bauer et al., 2010; Bankstahl et al., 2013). The signal obtained with such a tracer should be, under ideal conditions (no unspecific binding for example), proportional to the level of P-gp expression and, therefore, upregulation of P-gp expression should result in an increased signal, which would be easier to measure (reduced noise levels). So far, however, these tracers have shown to be substrates at tracer doses despite their known inhibitory nature at pharmacological doses as determined in in vitro assays (Luurtsema et al., 2009). It has been postulated that, at these tracer doses, the concentration of these compounds is too low to achieve full inhibition of P-gp function, leading to transport of tracer and a picomolar affinity would be required to measure P-gp expression (Kannan et al., 2013). This dose dependent behaviour has been confirmed for [^11^C]laniquidar in experiments with variable amounts of co-injected unlabelled laniquidar (Moerman et al., 2012). Clearly, this significantly limits the usefulness of this class of P-gp tracers for studying upregulated P-gp expression, since the ideal tracer to delineate P-gp expression is an inhibitor that binds to the protein but is not transported (Mullauer et al., 2013). On the other hand, if a P-gp inhibitor is not a substrate for P-gp at any dose, then a lower affinity would suffice.

Recently, in several in vitro assays, a large series of analogues of isatin thiosemicarbazides were identified as selective and potent compounds, which interact with P-gp (Hall et al., 2009). Within this series, 2-(5-fluoro-2-oxoindolin-3-ylidene)-N-(4-methoxyphenyl)hydrazine-carbothioamide was identified as an interesting lead compound for the development of a PET tracer that would selectively interact to P-gp without being transported, since its structure does not resemble any of the known P-gp inhibitors, it might have different characteristics toward P-gp (Hall et al., 2009). This interaction however, should be assessed in P-gp Knockout animals, whether this interaction is indeed towards P-gp. As the 5 position of the isatin moiety is not a suitable position for a nucleophilic aromatic fluorination and with the 4 and 6 positions only slightly activated, the purpose of the present study was to develop the synthesis of two analogues, [^18^F]2-(4-fluoro-2-oxoindolin-3-ylidene)-*N*-(4-methoxyphenyl)hydrazine-carbothioamide ([^18^F]**5**) and [^18^F]2-(6-fluoro-2-oxoindolin-3-ylidene)-*N*-(4-methoxyphenyl)-hydrazinecarbothioamide ([^18^F]**6**), and to assess their potency as P-gp tracers in vivo in rats and mice using both biodistribution and PET experiments.

## Methods

### General

Unless stated otherwise, chemicals were obtained from Sigma Aldrich (Zwijndrecht, the Netherlands) and used without further purification. Acetonitrile, methanol, ethyl acetate and hexane were purchased from Biosolve (Valkenswaard, the Netherlands), 4-nitroisatin (4-nitro-2,3-dioxyindole) and 6-nitroisatin (6-nitro-2,3-dioxyindole) from Bio-Connect BV (Huissen, the Netherlands), 4-(4-methoxyphenyl)-3-thiosemicarbazide (ABCR, Giessen, Germany) and tariquidar from AzaTrius Pharmaceuticals Pvt. Ltd. (Mumbai, India). [^18^F]F^−^ was produced using an IBA Cyclone 18/9 cyclotron. Radiosyntheses were performed using an in house built synthesis device (Windhorst et al., 2001).

Nuclear magnetic resonance (NMR) data were obtained using a Bruker AC 200 (Bruker, Billerica, USA) and chemical shifts (δ) were defined relative to the signal of the solvent (7.27 for CHCl_3_, 2.50 for DMSO-*d6*). The following high performance liquid chromatography (HPLC) methods were used: for preparative HPLC (method A) an ACE 5 μM C18, 250*10 mm column (Alltech, Breda, the Netherlands) with acetonitrile/50 mM ammonium dihydrogenphosphate in water (pH = 4.7) 50/50 (*v*/v) as eluent at a flow of 4 mL∙min^− 1^_,_ UV monitoring at 254 nm and radioactivity detection with an in house built radioactivity detector; for analytical HPLC (method B) a Kromasil 100 C18 10 μM, 250*10 mm (Alltech, Breda, the Netherlands) with acetonitrile/water/diisopropylamine 50/50/0.2 (v/v/v) as eluent at a flow of 1 mL∙min^− 1^, UV monitoring at 254 nm and a Raytest 2.5 in. NaI(Tl) radioactivity detector (Raytest, Straubenhardt, Germany); for metabolite analysis (method C) a Gemini C18, 5 μm 250*10 mm (Phenomenex, Utrecht, the Netherlands) with the following gradient: A = acetonitrile and B = 50 mM ammoniumacetate in water. *T* = 0 min: 0.25 mL·min^− 1^, *T* = 0.5 min: 6.00 mL·min^− 1^, *T* = 14.5 min: 6.00 mL·min^− 1^, *T* = 15.0 min: 0.25 mL·min^− 1^. Percentage of B: T = 0 min: 60%, *T* = 10 min: 30%, *T* = 13 min: 30%, *T* = 13.5 min: 60% and T = 15 min: 60%, UV monitoring at 230 nm and radioactivity detection with an in house built radioactivity detector.

Sprague-Dawley rats were obtained from Harlan (Horst, the Netherlands). Prior to experiments, rats were housed in groups of 4 per cage. FVB (Friend virus B-type) P-gp knockout (MDR1a/b (−/−)), FVB BCRP (Breast cancer restistant protein) knockout and FVB wild type mice were obtained from Taconic (Hudson, USA). Animals were kept at a constant temperature of 21 °C, a constant humidity of 50–60% and a 12 h light/dark cycle, in which lights were switched on at 8:00 a.m. Animals had unrestricted access to food (Teklad Global 16% Protein Rodent Diet, Harlan, Madison, WI, USA) and water. All animal experiments were performed in compliance with the Dutch law on animal experimentation. After a habituation period of approximately one week, PET, biodistribution or metabolite studies were performed.

### Synthesis of 4-fluoroisatin (3) and 6-fluoroisatin (4)

Both (**3**) and (**4**) were synthesized using the method of Zhang *et al (*Zhang et al., 2010*)*. 12.8 g (89.6 mmol) Na_2_SO_4_ and 1.87 g (11.3 mmol) chloral hydrate were dissolved in 50 mL H_2_O at 35 °C. Next, a solution of 1.13 g (10.2 mmol) 3-fluoroaniline (**1**) in 15 mL H_2_O and 2 mL of 37% HCl in H_2_O was added, followed by the addition of 2.24 g (34.8 mmol) hydroxylamine hydrochloride in 20 mL water. This solution was then heated to 90 °C for 2 h, and gently cooled to 50 °C over 1 h. The solution was filtered and the filtrate was washed 3 times with 15 mL water, and subsequently the filtrate was dried under vacuum to yield 1.41 g (7.8 mmol) 3- fluoroisonitroacetanilide N-(3-fluorophenyl)-2-(hydroxyimino)acetamide (**2**) in 76%.

Next, 8 mL of concentrated H_2_SO_4_ was heated to 60 °C and 1.41 g (7.80 mmol) of (**2**) was added portion wise to this solution during 45 min. The mixture was heated to 85 °C for 15 min and then cooled in an ice bath for 1 h. The mixture was poured into 35 mL water and the precipitate was filtered off after 2 h. This precipitate was dissolved in 50 mL ethyl acetate and washed 3 times with 40 mL H_2_O. The ethyl acetate layer was dried with Na_2_SO_4_, filtered and the ethyl acetate was evaporated under vacuum to yield a mixture of (**3**) and (**4**). Hereafter, both (**3**) and (**4**) were purified and separated using flash column chromatography over silica, hexane/ethyl acetate 6/1, with an Rf of 0.25 for (**3**) and 0.15 for (**4**), to yield a mixture of 0.063 g (0.38 mmol) of (**3**) 5% and 1.02 g (6.1 mmol) of (**4**) 79%.

(**3**): ^1^H-NMR (DMSO-d6) δ: 7.60 (1H, m, aromatic proton), 6.87 (1H, m, aromatic proton), 6.74 (1H aromatic proton) ^13^C-NMR (DMSO-d6) 182.8, 169.5, 167.4, 160.1, 153.8 d, 128.3 d, 115.3 d, 110.4 d, 110.2 d, 108.9, 100.7 d, and HRMS (ESI) m/z calculated for C_8_H_5_FNO_2_ (M^+^) 166.0299 found 166.0308.

(**4**): ^1^H-NMR (DMSO-d6) δ: 7.62 (1H, M, aromatic proton), 6.84 (1H, t, aromatic proton, *J =* 8 Hz), 6.72 (1H, d, aromatic proton, *J =* 8 Hz) ^13^C-NMR (DMSO-d6) δ: 180.4, 149.5, 159.2, 157.1, 151.8 d, 141.2 d, 110.4 d, 108.9, 106.5 d and HRMS (ESI) m/z calculated for C_8_H_5_FNO_2_ (M^+^) 166.0299 found 166.0307.

### Synthesis of (Z)-2-(4-fluoro-2-oxoindolin-3-ylidene)-N-(4-methoxyphenyl)hydrazine-carbothioamide (5)

50 mg (0.30 mmol) of (**3**) was dissolved in 15 mL ethanol. To this solution 64 mg (0.30 mmol) of 4-(4-methoxyphenyl)-3-thiosemicarbazide was added, as well as 20 μL of acetic acid. This solution was heated to 110 °C in a closed reaction vessel for 2 h, after which it was cooled to room temperature and left overnight so that (**5**) crystallized out of the ethanol. The product was washed twice with 15 mL cooled ethanol (4 °C) and dried under vacuum overnight to yield 59 mg (0.17 mmol) of (**5**) 57%.

(**5**): ^1^H-NMR (DMSO-d6) δ: 13.06 (1H, s, NH), 11.52 (1H, s, NH), 10.38 (1H, s, NH), 7.44–7.46 (3H, m, aromatic protons), 6.92–6.97 (3H, m, aromatic protons), 6.79 (1H, d, aromatic proton *J =* 4 Hz), 3.77 (3H, s, OCH_3_) ^13^C-NMR (DMSO-d6) δ: 179.2, 165.1, 160.0, 158.0, 146.5 d, 135.9 d, 134.0, 133.2 t, 129.7, 116.2, 112.6, 112.5, 110.2 d, 109.8, 109.7 58.0 and HRMS (ESI) *m/z* calcd for C_16_H_14_FSN_4_O_2_ 345.0816 (M^+^) found 345.0787.

### Synthesis of (Z)-2-(6-fluoro-2-oxoindolin-3-ylidene)-N-(4-methoxyphenyl)hydrazine-carbothioamide (6)

**Fig. 1:**
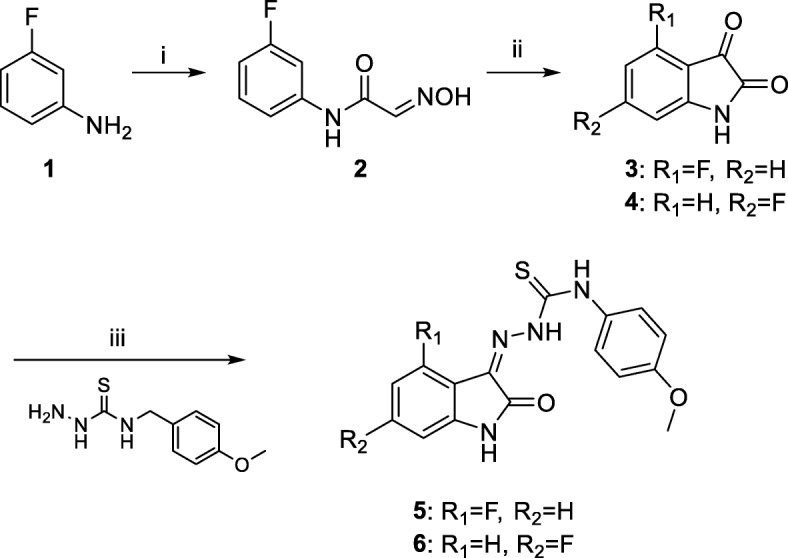
Reaction conditions: i: Na_2_SO_4,_ chloral hydrate_,_ H_2_O, HCl, hydroxylamine hydrochloride 90 °C 2 h; ii H_2_SO_4_ 85 °C 15 min; iii ethanol 110 °C 2 h

Starting with 50 mg (0.30 mmol) of (**4**), (**6**) was synthesized according to the same procedure as used for (**5**) to yield 69 mg (0.20 mmol) of (**6**) in 65% yield, and shown in Fig. 1.

(**6**): ^1^H-NMR (DMSO-d6) δ: 12.6 (1H, s, NH), 11.2 (1H, s, NH), 10.8 (1H, s, NH), 7.63 1H, m, aromatic proton), 7.46 (2H, d, aromatic protons, *J* = 8 Hz), 7.20 (1H, m, aromatic proton) 6.98 (2H, d, aromatic protons, *J* = 8 Hz), 6.94, (1H, m, aromatic proton), 3.79 (3H, s, OCH_3_), ^13^C-NMR (DMSO-d6) δ: 179.2, 165.5, 161.9, 160.1, 160.0, 141.4, 134.1 d, 133.8, 129.9, 124.1 d, 120.2 d, 116.3, 114.8 d, 110.9 d, 58.0 and HRMS (ESI) *m/z* calculated for C_16_H_14_FN_4_O_2_ 345.0816 (M^+^) found 345.0791.

### Radiosynthesis

[^18^F]**5** was synthesized starting from 15 to 74 GBq [^18^F]F^−^ in a mixture of 1 mL, 9/1 (*v*/v) acetonitrile/water, containing 13 mg (34.5 μmol) of kryptofix and 2 mg (14.5 μmol) of potassium carbonate. This mixture was dried under vacuum at 90 °C for 6 min, while passing a helium flow (50 mL·min^− 1^) over the solution. This was repeated with 1 mL of dry acetonitrile. Next, 0.5 mL of dry DMSO containing 4.6 mg (24 μmol) of 4- or 6-nitroisatin was added and the mixture was heated at 140 °C for 40 min. Then, 4.5 mL of water was added and the total was passed over a Waters tC-18 plus Sep-Pak, pre-treated with 5 mL of ethanol, followed by 5 mL of water. The Sep-Pak was rinsed with 10 mL of water and, using 1.5 mL of ethanol (98%), [^18^F]**3** was eluted from the Sep-Pak to a second reaction vial, which already contained 9 mg (42 μmol) of 4-(4-methoxyphenyl)-3-thiosemicarbazide. Next, 50 μL of acetic acid was added and the mixture was heated at 120 °C for 30 min. The solution was cooled to room temperature and quenched with 1.5 mL of water. This mixture was loaded onto HPLC and purified (method A). The product, [^18^F]**5** was eluted at 20 min and collected within 1 min. This fraction was diluted with 16 mL of water and passed over a Waters C18 light Sep-Pak cartridge, pre-treated with 5 mL of ethanol followed by 5 mL of water. Next, this cartridge was washed with 10 mL of water and reversely eluted with 2 mL of water containing 20 mass% Tween-80. The eluate was diluted with 2 mL of sterile saline and the total solution was passed over a Millex GV filter (Millipore, Amsterdam, the Netherlands). The radiochemical purity was determined by HPLC method B.

[^18^F]**6** was synthesized using exactly the same procedure as described for [^18^F]**5**, except that 6-nitroisatin was used instead of 4-nitroisatin as precursor for the radiosynthesis of [^18^F]**6**.

### Biodistribution

Biodistribution of both [^18^F]**5** (8.6 ± 1.8 MBq, mean ± SD) and [^18^F]**6** (6.8 ± 0.6 MBq) in Sprague-Dawley rats (*N* = 4 for both compounds) was determined at 10 and 45 min after injection. Animals were injected in a tail vein under isoflurane anaesthesia and euthanized at one of the two time points, again under isoflurane anaesthesia. Heart, lungs, liver, kidneys, testes and bone were dissected, and blood and urine were collected. In addition, olfactory bulb, hippocampus, striatum, cerebral cortex, cerebellum and remaining parts of the brain were dissected. All tissues were counted for radioactivity using a gamma counter (LKB Wallac, Turku, Finland) and weighed. Results were expressed standard uptake value (SUV).

### Metabolite analysis

Metabolic profiles of both radiotracers in both plasma and brain were assessed in Sprague-Dawley rats, at 10 and 45 min after injection of the radiotracer (2 animals per time point). Blood (2 mL) was collected and half the brain was isolated during the biodistribution experiments, as described in the biodistribution section.

Blood was collected in heparin tubes and subsequently plasma was separated from cells by centrifugation for 5 min at 4000 rpm (Hettich universal 16, Depex B.V., Houten, the Netherlands). Thereafter, plasma was loaded onto a tC18 Sep-Pak (Waters, Etten-Leur, the Netherlands), washed with 3 mL of water, and the eluate was considered to be the radiolabelled polar metabolite fraction, which was confirmed using HPLC method C. Next, the Sep-Pak was eluted with 2 mL of methanol to obtain the radiolabelled non-polar metabolite fraction. This fraction was analysed using HPLC method C.

Brain tissue was homogenised using an ultrasonic homogenizer (Braunsonic 1510, Germany) in water, under ice cooling, and subsequently centrifuged at 4000 rpm for 5 min. Separated supernatants were loaded onto a tC18 Sep-Pak, washed with 3 mL water to obtain the polar fraction and subsequently washed with 2 mL methanol to obtain the non-polar fraction, which again was analysed using HPLC method C.

### PET studies

Animals were anaesthetized via a nose mask with 4 and 2% isoflurane in oxygen for induction and maintenance, respectively, at a rate of 1 L·min^− 1^. One hour prior to each study, a femoral vein and a femoral artery were cannulated for administration of radiotracer and tariquidar, and for blood sampling, respectively. In mice no blood sampling was performed. Blood oxygen saturation and body temperature were monitored continuously throughout surgery and experiments. Heating and oxygen supply were adjusted to maintain a body temperature of ~ 36 °C and an oxygen saturation > 96%.

Rats were positioned in pairs, while mice were scanned in groups of 5 using a double LSO/LYSO layer High Resolution Research Tomograph (HRRT; Siemens/CTI, Knoxville, TN, USA) (Zhang et al., 2010). First, for attenuation and scatter correction purposes, a transmission scan was acquired using a 740 MBq 2-dimensional (2D) fan-collimated ^137^Cs (662 keV) moving point source (van Velden et al., 2008; de Jong et al., 2007).

**Scheme 1:**
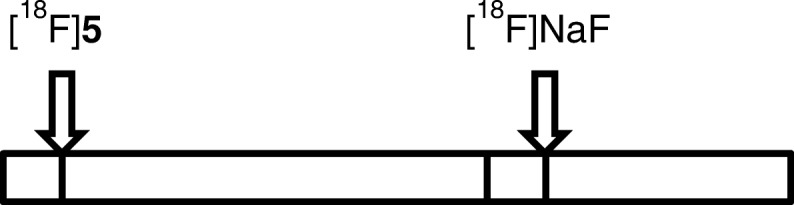
showing 5 min. Transmission scan, followed by injection of [^18^F]**5** during a 60 min scan, after which a second transmission scan was performed. Next [18F]NaF was injected during a 30 min. Scan

Immediately after the transmission scan, [^18^F]**5** (6.3 ± 0.7 MBq (350–400 μL) for rats, 2.2 ± 0.3 MBq (120–150 μL) for mice was administered and a dynamic emission scan of 60 min was started. In the case of rat studies, blood samples were withdrawn at ten time points during the scan, weighed and counted for amount of radioactivity. At the end of this emission scan, for both rats and mice [^18^F]NaF (3.6 ± 0.4 MBq) was injected and a second emission scan was initiated (30 min). This last scan was used for co-registration with an MR based rat brain atlas (Buiter et al., 2012), while for the mice study the co-registration of the brain was performed manually using the [^18^F]NaF scan as a reference for the skull as shown in Scheme 1.

After the [^18^F]NaF scan, both cannulation lines were filled with a mixture of Polyvinylpyrrolidonum / heparin in saline and closed. Next, animals were allowed to recover from anaesthesia and kept overnight with free access to food and water. The following day, rats were treated with 15 mg/kg tariquidar (Bankstahl & Lösscher, 2008) *iv,* 15 min prior to an identical imaging procedure. On the second day the dose of [^18^F]**5** was 6.8 ± 0.3 MBq and the dose of [^18^F]NaF 4.9 ± 0.3 MBq.

Because [^18^F]**5** and [^18^F]**6** were formulated in a 10% tween-80 containing solution, its effect on P-gp function was also investigated. In a separate group of rats (*N* = 4) (*R*)-[^11^C]verapamil data were obtained. After a transmission scan, a dynamic emission scan (60 min) was acquired immediately following administration of (*R*)-[^11^C]verapamil (11.3 ± 1.2 MBq) (450-500 μl) formulated in 10 mass% of Tween-80. After the baseline scan, animals were treated with 15 mg/kg tariquidar *iv*. Fifteen minutes after tariquidar administration,).: a second emission scan was acquired after injection of (R)-[^11^C]verapamil (6.4 ± 2.5 MBq) formulated in 10 mass% of Tween-80 scan, followed by a third 30-min emission scan was acquired after injection of [^18^F]NaF (6.0 ± 0.5 MBq).

List mode data of both acquired [^18^F]**5** and (*R*)-[^11^C]verapamil scans were rebinned into the following frame sequence: 7 × 10, 1 × 20, 3 × 30, 2 × 60, 2 × 150, 2 × 300 and 4 × 600 s. The [^18^F]NaF scan was rebinned into a single 1800 s frame. Following normalization of the data and corrections for decay, dead time, attenuation, randoms and scatter, scans were reconstructed using 3D ordinary Poisson ordered subsets expectation maximization. This resulted in images with an average spatial resolution of 2.5 mm full width at half maximum (van Velden et al., 2009).

Image data were analysed using the freely available software package Amide 0.9 (Loening & Gambhir, 2003). An MR based rat brain atlas was used to define a whole brain region of interest. For each rat, the MR atlas was aligned visually with the [^18^F]NaF image using a procedure described previously (Buiter et al., 2012).

## Results

### Chemistry

(**3**) and (**4**) were synthesized in overall yields of 4 and 60%, respectively, whilst the last synthesis steps of the reference compounds for (**5**) and (**6**) were obtained with yields of 57 and 65%, respectively. This led to an overall synthesis yield of 2.1% for (**5**) and 39% for (**6**). After purification, both reference compounds (**5**) and (**6**) showed no impurities and they were analysed using proton and carbon NMR. Exact masses were found to be 345.0787 for (**5**) and 345.0791 for compound (**6**).

### Radiosynthesis

**Fig. 2:**
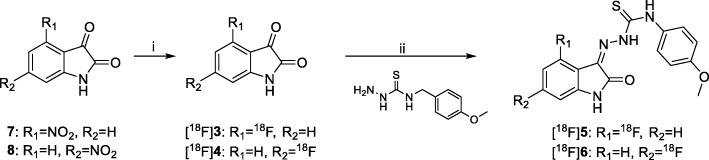
reaction conditions: (i) DMSO, [^18^F]fluoride, Kryptofix[2.2.2], K_2_CO_3_, 40 min. 140 °C (5–6% yield) (ii) ethanol, acetic acid, 30 min. 120 °C (35–50% yield)

Radiosynthesis of [^18^F]**3** was optimized according to Table 1. The highest yields were obtained when performing the reaction in DMSO at 140 °C for 40 min with 24 μmol of 4-nitroisatin.These optimized conditions were also used for the synthesis of [^18^F]**4**. The reaction scheme is shown in Fig. 2.

**Table 1:** Optimization of reaction conditions for radiosynthesis of [^18^F]**3**

Solvent 0.5 mL	Precursor (μmol)	Temperature (°C)	Kryptofix (μmol)	K_2_CO_3_ (μmol)	Yield (%)
DMSO	26	120	6	3	6%
DMSO	24	140	6	3	7%
DMSO	54	140	6	3	5%
DMSO	26	160	6	3	4%
MeCN	26	140	6	3	1%
MeCN	24	140	18	9	2%
DMSO	16	140	6	3	3%

Using a waters tC18 plus Sep-Pak it was possible to trap all [^18^F]**3** and, after elution with 1.5 mL ethanol, less than 1% of the total radioactivity remained on the cartridge. The yield of the second step was optimized to 35–50% using 9 mg (42 μmol) of 4-(4-methoxyphenyl)-3-thiosemicarbazide at 120 °C for 30 min in ethanol. The same reaction conditions were used in the radiosynthesis of [^18^F]**6**.

Formulation of the final product was performed with a Waters tC-18 light Sep-Pak. After dilution with water, the product was trapped, washed with 10 mL of water and then eluted with 2 mL of 20 mass% Tween-80 in water. Approximately 10–20% of the radioactivity remained on the Sep-Pak. After dilution with 2 mL water, the product was passed over a Millex GV sterile filter, yielding 2.1 ± 0.9% of [^18^F]**5** (*N* = 7) or 2.3 ± 1.0% of [^18^F]**6** (*N* = 6), both corrected for decay and with a specific activity of 91 ± 23 GBq·μmol^− 1^ for [^18^F]**5**, and 97 ± 25 for [^18^F]**6** at end of synthesis.

Both [^18^F]**5** and [^18^F]**6** did not appear to be stable in either ethanol or saline (Table 2), Moreover, addition of gentisic acid or ascorbic acid as radical scavengers were also tested to enhance the stability in the product vial. Using DMSO instead of ethanol did not result in a stable product either. Both [^18^F]**5** and [^18^F]**6** were stable within a range of 10% -100% acetonitrile in water (not shown in Table 2), as well as in a solution of 2.5% and 20% Tween-80 in saline and without the use of ethanol.

**Table 2:** Stability of [^18^F]**5** assessed in several formulation solutions; *N* = 2 for each time point

Time (Minutes)	5	15	30	45	60	90
10% ethanol in saline	92	–	–	–	76	69
DMSO	89	85	–	–	72	–
Ethanol	97	80	–	67	–	–
Free radical scavengers	95	–	89	–	79	–
acetonitrile	96	–	96	–	96	–
20% Tween-80 in saline	95	95	–	95	–	–
2.5% Tween-80 in saline	95	94	94	94	94	–

### Biodistribution

**Fig. 3:**
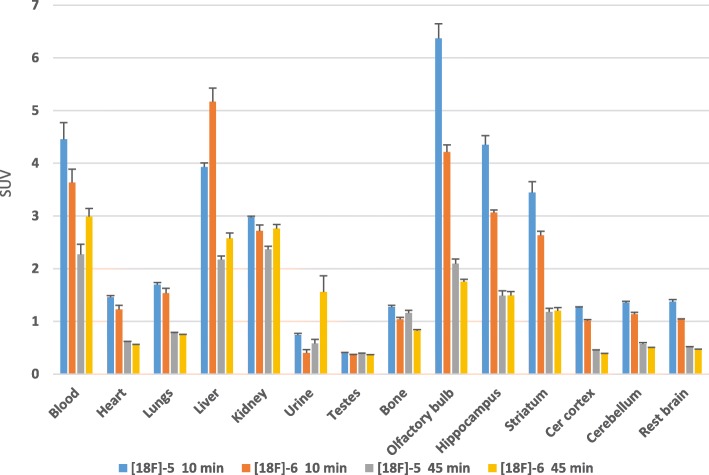
Biodistribution of [^18^F]**5** and [^18^F]**6** in Sprague-Dawley rats (*N* = 4) at 10 and 45 min after injection depicted in SUV

As shown in Fig. 3 the concentration of radioactivity in blood remained relatively high with SUV values at 45 min of about 2 and 3 for [^18^F]**5** and [^18^F]**6**, respectively. The amount of activity in peripheral organs was relatively low with the highest uptake in the liver with an SUV at 10 min of 3.9 and 5.2 for [^18^F]**5** and [^18^F]**6**, respectively. Uptake in the testes was relatively low (SUV between 0.2 and 0.3 for [^18^F]**5** and [^18^F]**6**, respectively), being rather stable between 10 and 45 min after injection. Bone uptake of both tracers appeared to decrease slowly over time. All brain areas showed high uptake of both tracers together with moderate washout. The highest uptake in the brain was seen in olfactory bulb, hippocampus and striatum, whilst cerebral cortex, cerebellum and remaining parts of the brain showed lower brain uptake (Fig. 3).

### Metabolite analysis

Metabolite analysis, performed in Sprague Dawley rats (*N* = 2 for each tracer), showed rapid metabolism of both tracers, especially for [^18^F]**6** (Table 3). As early as 10 min after injection, most of the radioactivity in plasma was due to radiolabelled metabolites. For [^18^F]**6**, high levels of radiolabelled metabolites (~ 80%) in the brain were detected. The level of radiolabelled metabolites in the brain was lower for [^18^F]**5.** At 45 min after injection, 68% of the radioactivity in the brain was in the form of intact [^18^F]**5**.

**Table 3:** Percentage of intact tracer in plasma and brain after 10 and 45 min (*N* = 2)

	% intact [^18^F]5	% intact [^18^F]6
10 min		
Plasma	21.5 ± 0.7	14.0 ± 2.8
Brain	62.5 ± 6.4	20.5 ± 0.7
45 min		
Plasma	11.5 ± 2.1	7.0 ± 0.0
Brain	68.5 ± 2.1	16.0 ± 0.7

**Fig. 4:**
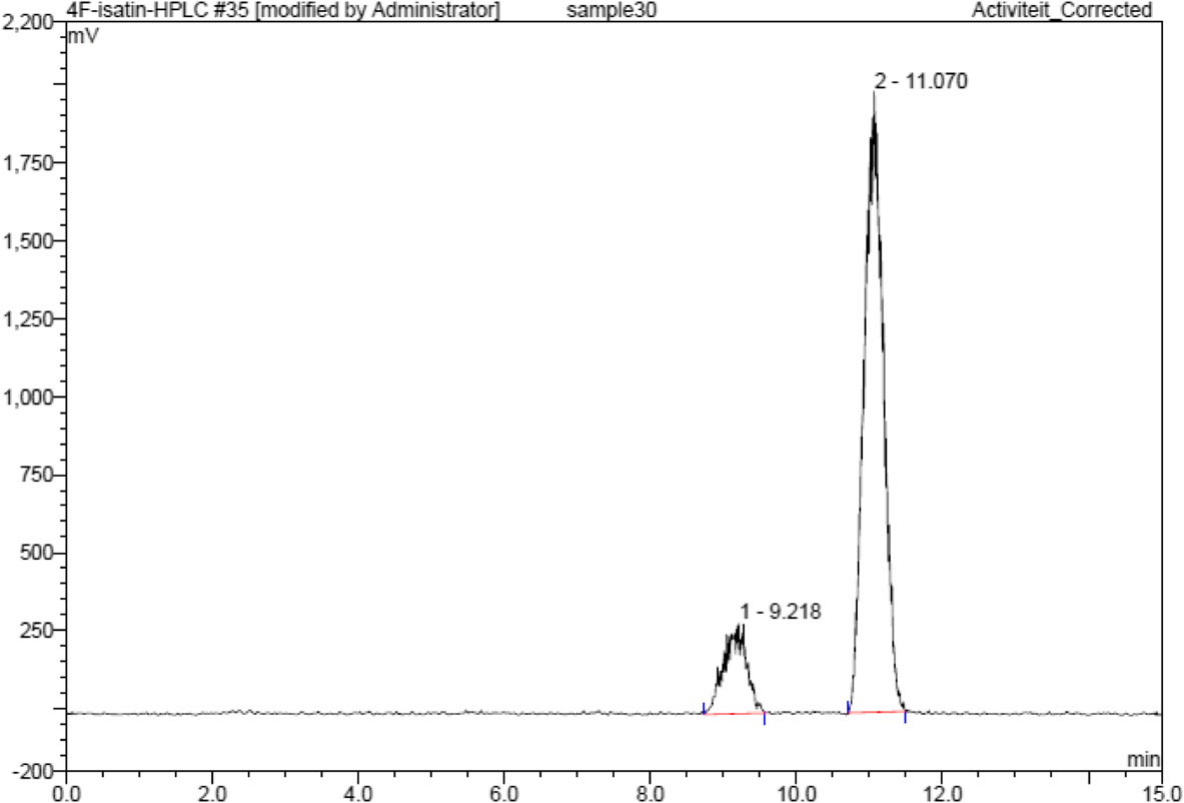
A representative HPLC chromatogram (method C) of the non-polar fraction of the brain homogenate at 10 min after injection, showing both [^18^F]**5** (Rt 11 min) and a labelled metabolite (Rt 9 min)

In the brain, only one main metabolite was observed for both [^18^F]**5** and [^18^F]**6**. A representative HPLC chromatogram of [^18^F]**5** is shown in Fig. 4, indicating an unidentified metabolite eluting 2 min before [^18^F]**5**. In plasma, however, metabolites were mainly found in the polar fraction, although the brain metabolite was also observed.

### PET studies

#### Effect of Tween-80 on uptake of (R)-[^11^C]verapamil

**Fig. 5:**
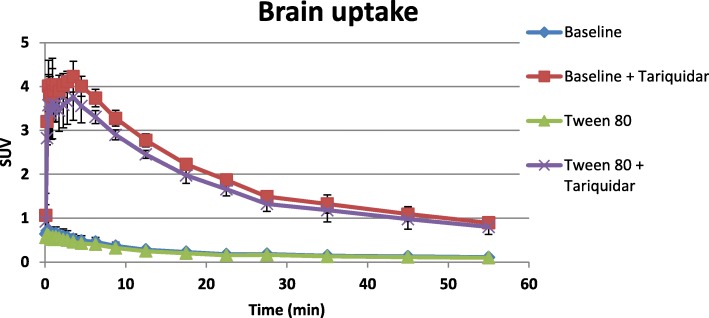
Cerebral uptake of (R)-[^11^C]verapamil in naïve and 15 mg/kg tariquidar pre-treated rats, both with and without formulation in 10% Tween-80; data without 10% Tween-80 were derived from Syvänen et al *(Syvänen et al.,* 2011*)*

Since both [^18^F]**5** and [^18^F]**6** were formulated in a solution containing 10% tween-80, the effect of 10% Tween-80 on cerebral uptake of (*R*)-[^11^C]verapamil was investigated. In Fig. 5, uptake of (*R*)-[^11^C]verapamil formulated with 10% Tween 80 (*N* = 4) is shown both before and after treatment with 15 mg/kg tariquidar. For comparison, previous data by Syvänen *et al (*Bankstahl & Lösscher, 2008*)*, in which (*R*)-[^11^C]verapamil was formulated in 10% ethanol, are also included. For both naïve and treated animals, 10% Tween-80 formulated (*R*)-[^11^C]verapamil showed a slightly higher, although insignificantly initial uptake of (*R*)-[^11^C]verapamil in the brain followed by faster clearance from the brain.

#### [^18^F]**5** uptake before and after tariquidar treatment

**Fig. 6:**
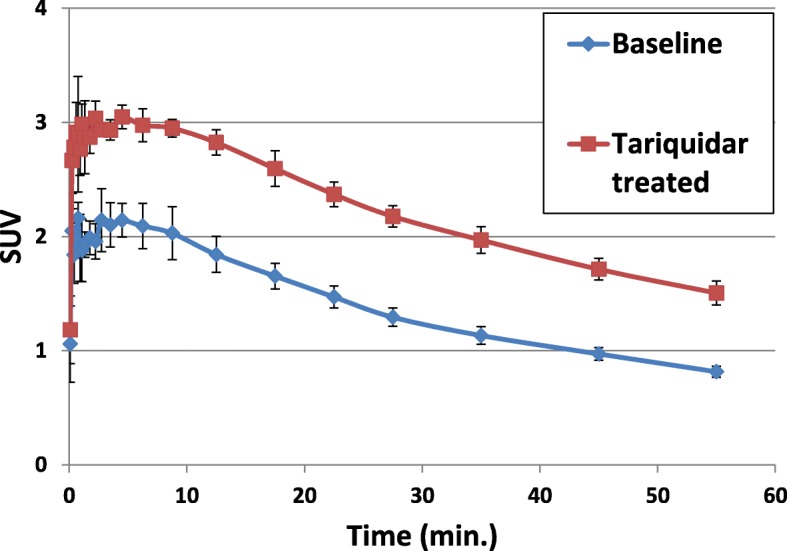
Whole brain time activity curves of [^18^F]**5** before (blue diamonds) and after (red squares) 15 mg/kg tariquidar (a specific P-gp inhibitor) treatment

**Fig. 7:**
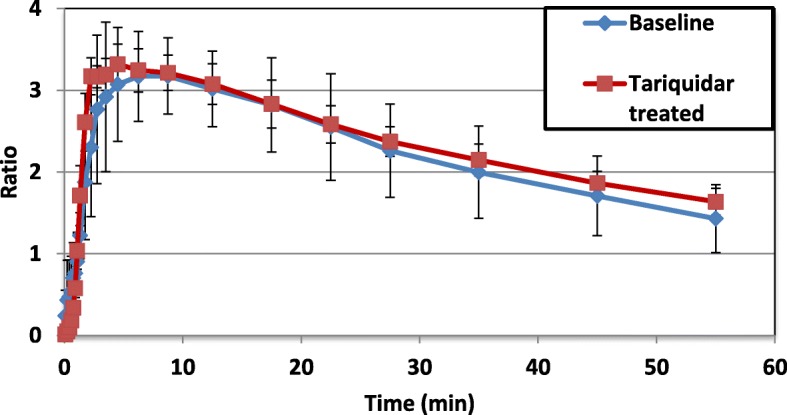
Brain to blood concentration ratio of [^18^F]**5** before (blue diamonds) and after (red squares) 15 mg/kg tariquidar

Whole brain time activity curves of [^18^F]**5** (N = 4) are shown in Fig. 6. Maximum SUV was, on average, 2.2 at 5 min, followed by slow washout to a level of 0.8 after 45 min. Both brain and blood concentrations increased after tariquidar treatment. Brain SUV increased to about 3.0 and 1.5 at 5 and 45 min, respectively, whilst blood concentrations increased by a factor of 1.5 ± 0.2 after 45 min. As a first order correction for increased delivery the ratio of brain SUV to whole blood SUV was calculated. This ratio was not significantly different between baseline and tariquidar pretreated scans (Fig. 7).

#### [^18^F]**5** uptake in WT, P-gp and BCRP knockout mice

**Fig. 8:**
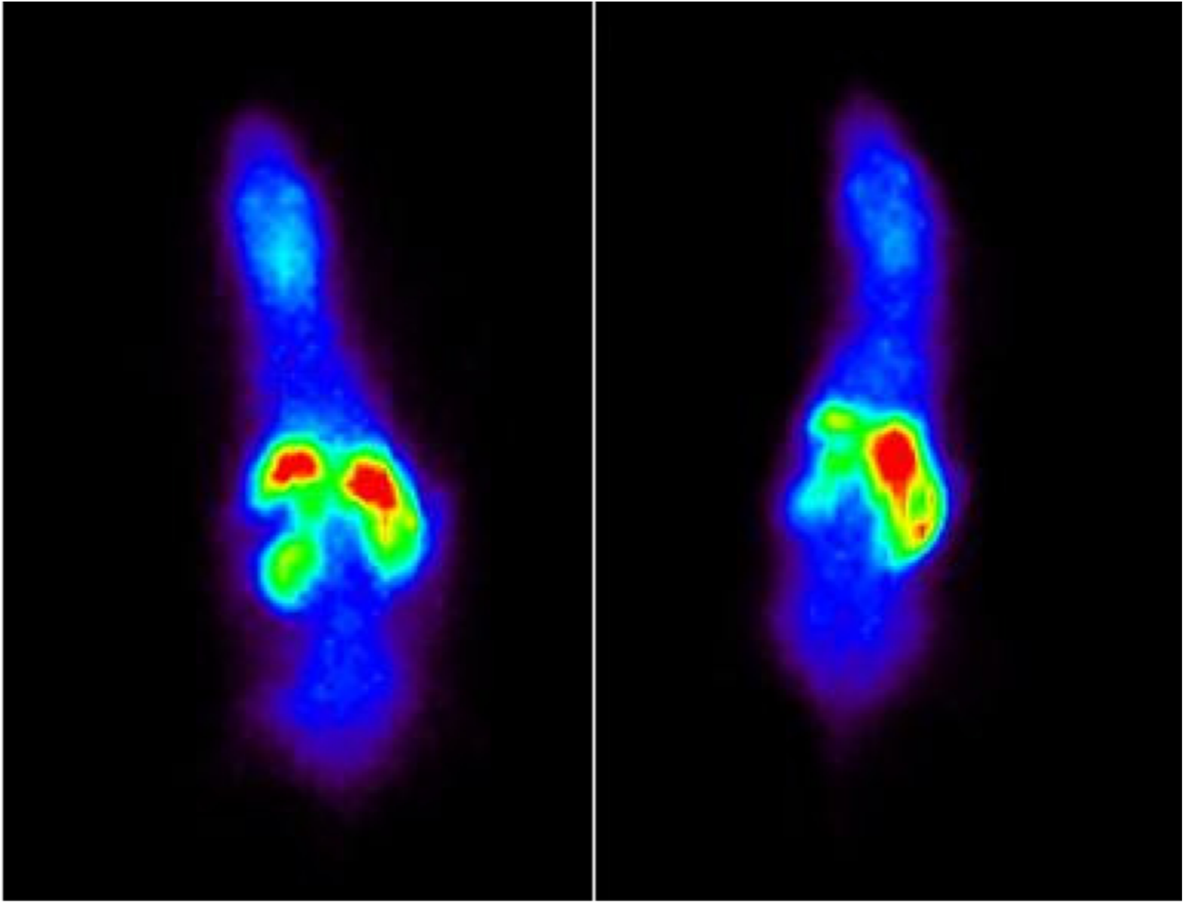
Summed PET image (2–20 min) of [^18^F]**5** in a wild-type mouse (left) and a P-gp KO mouse (right)

**Fig. 9:**
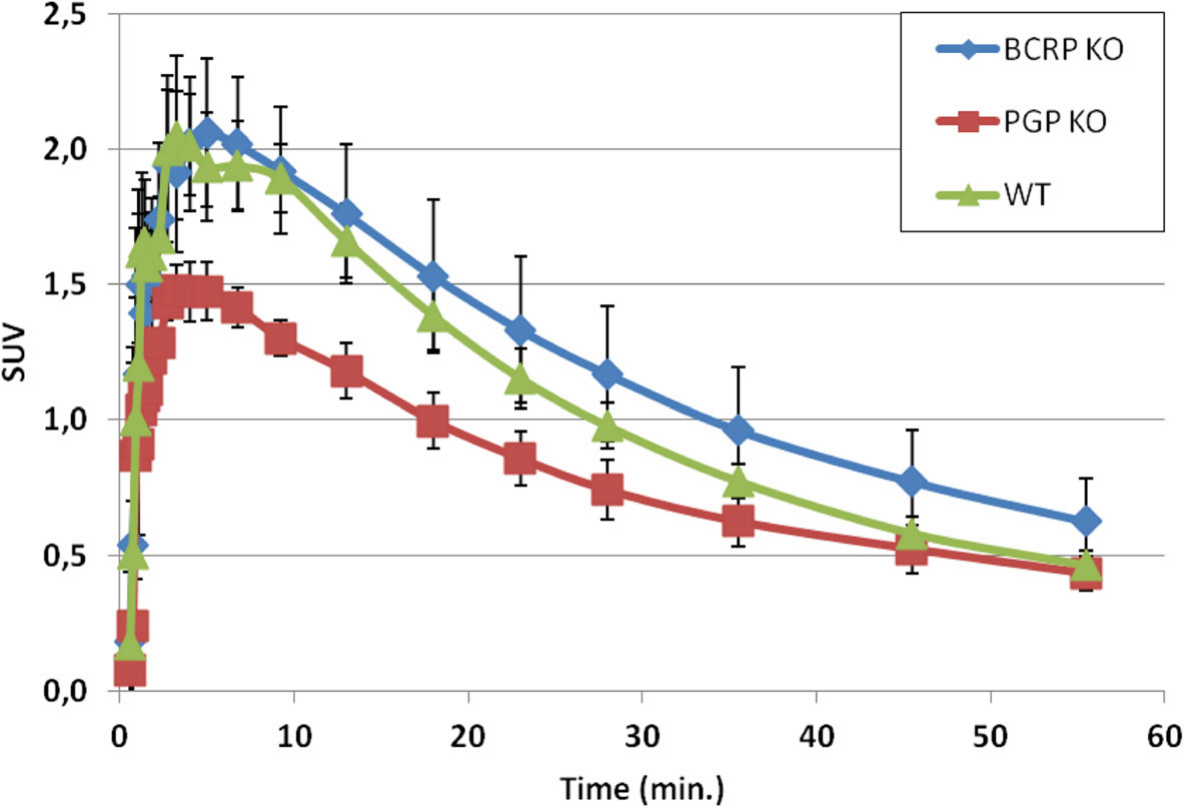
Whole brain time activity curves of [^18^F]**5** in P-gp KO mice (red squares), BCRP KO mice (blue diamonds) and WT mice (green triangles)

PET studies were performed in both P-gp and BCRP knockout mice, and results were compared with those in wild-type mice. Ten minutes after injection, brain SUV of [^18^F]**5** was 1.3 ± 0.1 (N = 4), 1.9 ± 0.2 (*N* = 5) and 1.9 ± 0.1 (N = 5) for P-gp knockout, BCRP knockout and wild type mice, respectively. A summed PET image (2–20 min) of [^18^F]**5** in a WT mouse and a P-gp KO mouse is shown (Fig. 8). A significant reduction in brain uptake compared with wild type mice, with a *P* < 0.05 was observed between 5 and 28 min, differences in uptake between mice lost its significance at later time points (Fig. 9).

**Fig. 10:**
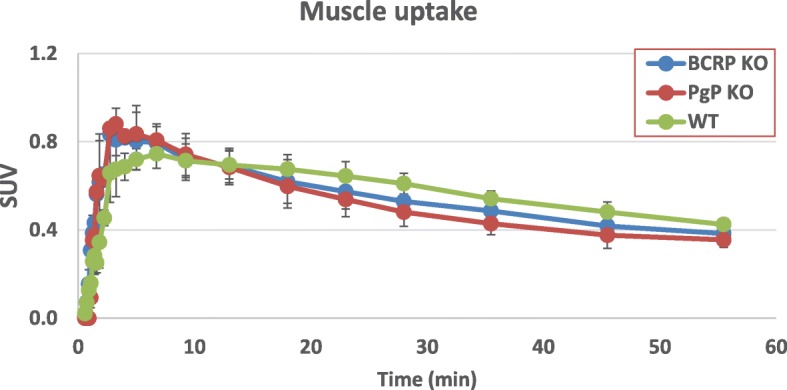
Muscle time activity curves of [^18^F]**5** in P-gp KO mice (red squares), BCRP KO mice (blue diamonds) and WT mice (green triangles)

Because of the low blood volume of mice, no blood samples were withdrawn. In addition, no image derived input function could be obtained due to the relatively low resolution of the HRRT scanner in comparison with the size of the mouse heart. To assess whether there were any differences in delivery between groups, uptake in muscle was also measured. The muscle uptake in Fig. 10 shows the uptake of [^18^F]**5** depicted in SUV in the 2 KO mice compared to the WT mice, and the uptake appears to be similar for all three strains, thus indicating that there are no differences in blood concentration, since the uptake is mainly dependent on influx of blood flow.

## Discussion

2-(5-fluoro-2-oxoindolin-3-ylidene)-N-(4-methoxyphenyl)hydrazine-carbothioamide was, despite its micromolar IC_50_, identified as an interesting lead for the development of an F-18 labelled PET ligand, which would selectively bind to P-gp without being transported (Hall et al., 2009). Unfortunately, it was not possible to synthesize [^18^F]5-fluoroisatin, presumably due to the position of the leaving group on isatin that cannot be properly activated to allow for a nucleophilic aromatic substitution reaction with [^18^F]fluoride. Hall et al. (Hall et al., 2009), however, have shown that modifications at this part of the isatin moiety are allowed without significantly affecting the inhibitory potency towards P-gp. Therefore, it was assumed that altering the structure by introducing an F-18 label at either the 4 or 6 position of isatin, would only have a minor effect on its inhibitory potency. In addition, in vitro assays have shown limited predictive value for P-gp binding at tracer levels and, therefore, the affinity for P-gp was assessed directly in a knockout animal model.

4-Fluoroisatin **3** and 6-fluoroisatin **4** were synthesized in an overall yield of 4 and 60%, respectively. These yields were slightly lower than those reported by Zhang *et al (*Zhang et al., 2010*)*, which may be due to the small scale synthesis performed in the present study as crystallization usually performs better at a larger scale. The synthesis yields of the two isatin-β-thiosemicarbozones were also comparable with those reported previously (Hall et al., 2009) for the 5-fluoro isomer. This led to an overall synthesis yield of 2.1% for (Z)-2-(4-fluoro-2-oxoindolin-3-ylidene)-N-(4-methoxyphenyl)hydrazinecarbothioamide **5** and 39% for (Z)-2-(6-fluoro-2-oxoindolin-3-ylidene)-N-(4-methoxyphenyl)hydrazinecarbothioamide **6**, which was sufficient for synthesis of the reference compounds.

The first step in the radiosynthesis of [^18^F]**3** was optimized by varying several parameters, such as amount of precursor, time, temperature and solvent. This led to an optimized radiochemical yield of 5–7%, which makes this step the limiting step regarding the overall yield, these optimized conditions were also used for the synthesis of [^18^F]**4**. This low yield can be expected based on the poorly activated labelling positions on the aromatic ring of both 4-nitroisatin and 6-nitroisatin.

Radiosyntheses of [^18^F]**5** and [^18^F]**6**, starting from [^18^F]**3** and [^18^F]**4**, respectively, were optimized by changing temperature and reaction time to obtain a yield of 35–50% in 1.5 mL of ethanol, 50 μL acetic acid, and 9 mg (42 μmol) of 4-(4-methoxyphenyl)-3-thiosemicarbazide for 30 min at 120 °C in a closed reaction vial. Reducing reaction time or amount of the carbazide, or changing the reaction temperature led to lower yields. Both acetonitrile and DMSO were also considered as solvent, but this led to a decrease in yield. The large amount of 4-(4-methoxyphenyl)-3-thiosemicarbazide was required due to the fact that unreacted nitroisatin precursor could not be separated from [^18^F]**3** by the Sep-Pak procedure. Consequently, 26 μmol nitroisatin was still present during the second reaction, which also reacted with 4-(4-methoxyphenyl)-3-thiosemicarbazide.

Both [^18^F]**5** and [^18^F]**6** did not appear to be stable in either ethanol or saline (Fig. 3), which is a major drawback for future applications, since most PET tracers are reformulated in concentrations of up to 10% of ethanol in saline of buffer. Moreover, addition of gentisic acid or ascorbic acid as radical scavengers (Table 2), did not improve stability of the formulated product. Which is indicating that radical formation might not be the cause of the instability of [^18^F]**5** and [^18^F]**6**. Both [^18^F]**5** and [^18^F]**6** were stable within a range of 10% -100% acetonitrile in water, but these amounts of acetonitrile cannot be tolerated for iv injection. Finally, after assessing the formulation solution within a range of 2.5% - 20% Tween-80 in water and without using ethanol, both [^18^F]**5** and [^18^F]**6** were stable over time. A solution of up to 10% Tween-80 can be used in animal studies (Li & Zhao, 2007). As an alternative to the Sep-Pak method, evaporation was performed by passing a flow of helium gas through the solution at room temperature, but this procedure led to decomposition of the radiotracers, which in turn led to the conclusion that a Sep-Pak procedure was required, and thus the ethanol, to reformulate both [^18^F]**5** and [^18^F]**6**.

Although the yield of the formulated product was quite low (2% to 3%), the synthesis was reliable and produced adequate amounts of product for biological evaluation. No failures were observed during 16 syntheses.

Biodistributions of [^18^F]**5** or [^18^F]**6** were assessed in Sprague-Dawley rats at 10 min (*N* = 4) and 45 min (N = 4) after radiotracer injection. At 45 min, radioactivity levels in blood remained relatively high for both tracers. In contrast, radioactivity in peripheral organs was relatively low, indicating fast washout. As bone uptake of both [^18^F]**5** and [^18^F]**6** was low and even decreased over time, defluorination of the tracers seems unlikely and PET measurements of cerebral uptake will not be confounded by high uptake in the skull. Baseline uptake in the testes (0.15% ID/g) was also increased compared with (*R*)-[^11^C]verapamil (Elsinga et al., 1996; Luurtsema et al., 2005) and [^11^C]tariquidar (Bauer et al., 2010), where the amount of activity in the testes remained below 0.3% ID/g. This is an indication of binding to P-gp in the blood-testes barrier as well. Both [^18^F]**5** and [^18^F]**6**showed high uptake in the brain with moderate washout.

Metabolism of both radiotracers was fast, especially that of [^18^F]**6**, which showed only 20% of intact compound in the brain already at 10 min. Metabolically, compound [^18^F]**5** was more stable with 63% intact tracer in the brain at 10 min. Based on the lower level of radiolabelled metabolites in the brain, [^18^F]**5** was pursued in in vivo animal studies, although 30–40% of metabolite fraction in the brain still is rather high.

Since both [^18^F]**5** and [^18^F]**6** were formulated in a 10% Tween-80 solution, it was necessary to assess the effects of Tween-80 on P-gp function. Therefore, PET data were acquired using (*R*)-[^11^C]verapamil, formulated in 10% Tween-80 in saline, comparing results with previously acquired PET data using (*R*)-[^11^C]verapamil formulated in 10% ethanol in saline, as reported by Syvänen et al. (Syvänen et al., 2011). As can be seen in Figs. 5, 10% Tween-80 only had a minor effect on final [^11^C]verapamil uptake, although initial brain uptake appeared a little higher in the first 5 min after injection.

In order to assess whether [^18^F]**5** is a P-gp substrate, a blocking study was performed using 15 mg/kg tariquidar, which is sufficient to completely block P-gp function (Syvänen et al., 2011). In the blocking study, a 20% increase in [^18^F]**5** uptake in the brain was observed. At the same time, however, radioactivity levels in whole blood also increased. Therefore, the ratio of brain SUV to blood SUV was used as a first order correction for increased delivery of [^18^F]**5** to the brain following tariquidar treatment. As shown in Fig. 9, the increased brain uptake could be explained entirely by increased delivery of [^18^F]**5**. This in turn was likely due to a blocking effect on peripheral binding sites. For comparison, brain uptake for the substrate tracer (*R*)-[^11^C]verapamil increased 10-fold following tariquidar treatment (Loening & Gambhir, 2003). In contrast, for [^18^F]**5**, it only increased by 30% (not corrected for blood input). These findings suggests that [^18^F]**5** is not a substrate for P-gp, and perhaps binds on or interacts with a different binding site, this however, should be further studied.

PET studies of P-gp knockout mice showed a significant reduction in brain uptake compared with wild type mice, with a *P* < 0.05 between 5 and 28 min thus supporting the observation that [^18^F]**5** binds to P-gp. At late times, time activity curves were not significantly different between knockout and wild type mice, most likely due to the large metabolite fraction. The uptake in the early time points confirm the in vitro findings by Hall *et al (*Hall et al., 2009*)*, indicating that the 5-fluoro analogue is a selective inhibitor of P-gp. Given these results, it seems very likely that [^18^F]**5** binds to P-gp, but is not a substrate for P-gp. Since pre-treatment with tariquidar gave similar PET results compared to baseline it is likely that [^18^F]**5** binds to a different binding site of P-gp than tariquidar or that an higher specific activity of [^18^F]**5** would be required, since the dose administered was in the range of 1.0 ± 0.2 pmol/gram. Further studies, such as metabolite analysis in knock out animals, are required to investigate this hypothesis in-depth.

Due to the limited blood volume of mice, blood concentrations in mice where not measured during the scanning procedure. Therefore, uptake in muscle was assessed and, as shown in Fig. 10, there were no significant differences observed between the different groups of animals. As there are no known binding sites in muscle, the similarity in muscle uptake between groups suggests that there are no significant differences in delivery. This, in turn, indicates that the differences in brain uptake between the different groups are due to differences in P-gp expression and not due to differences in delivery.

Finally, BCRP knockout mice showed no significant differences in brain uptake compared with wild type mice, indicating that [^18^F]**5** is neither binding nor being transported by BCRP in contrast to [^11^C]Elacridar for example (Bankstahl et al., 2013). Further (in vitro) studies are required to validate this compound as a P-gp expression tracer, for example performing the PET experiment in P-g knockout rats, which allow for blood sampling and metabolite analysis during the PET scan, which would also allow for reliable kinetic modelling. In addition a self-block, or an evaluation of compound [^18^F]**5** in the tumour models described by Hall *et al (*Hall et al., 2009*)*, might provide some extra additional information about the potency of compound [^18^F]**5**, this is however beyond the scope of the initial preclinical evaluation of [^18^F]**5**, which appears to be an interesting lead.

## Conclusion

Both [^18^F]**5** or [^18^F]**6** were successfully and reproducibly synthesized. Especially [^18^F]**6** showed fast metabolism. [^18^F]**5** appears to be a radiotracer that binds to P-gp, as showed in P-gp knock-out animals, but is not a substrate for P-gp. Therefore, it is a promising tracer for assessing P-gp expression levels in vivo*,* which however needs to be further investigated.
